# Comprehensive phylogeographic and phylodynamic analyses of global Senecavirus A

**DOI:** 10.3389/fmicb.2022.980862

**Published:** 2022-09-29

**Authors:** Han Gao, Yong-jie Chen, Xiu-qiong Xu, Zhi-ying Xu, Si-jia Xu, Jia-bao Xing, Jing Liu, Yun-feng Zha, Yan-kuo Sun, Gui-hong Zhang

**Affiliations:** ^1^Guangdong Provincial Key Laboratory of Zoonosis Prevention and Control, College of Veterinary Medicine, South China Agricultural University, Guangzhou, China; ^2^Maoming Branch, Guangdong Laboratory for Lingnan Modern Agriculture, Guangzhou, China; ^3^Key Laboratory of Animal Vaccine Development, Ministry of Agriculture and Rural Affairs, Guangzhou, China; ^4^National Engineering Research Center for Breeding Swine Industry, South China Agricultural University, Guangzhou, China; ^5^Guangdong Animal Health and Quarantine Office, Guangdong Animal Disease Prevention and Control Center, Guangzhou, China

**Keywords:** Senecavirus A (SVA), global dispersal, phylogeography, phylodynamics, phylogeny

## Abstract

Senecavirus A (SVA) is a member of the genus *Senecavirus* in the family Picornaviridae that infects pigs and shows symptoms similar to foot and mouth diseases and other vesicular diseases. It is difficult to prevent, thus, causing tremendous economic loss to the pig industry. However, the global transmission routes of SVA and its natural origins remain unclear. In this study, we processed representative SVA sequences from the GenBank database along with 10 newly isolated SVA strains from the field samples collected from our lab to explore the origins, population characteristics, and transmission patterns of SVA. The SVA strains were firstly systematically divided into eight clades including Clade I–VII and Clade Ancestor based on the maximum likelihood phylogenetic inference. Phylogeographic and phylodynamics analysis within the Bayesian statistical framework revealed that SVA originated in the United States in the 1980s and afterward spread to different countries and regions. Our analysis of viral transmission routes also revealed its historical spread from the United States and the risk of the global virus prevalence. Overall, our study provided a comprehensive assessment of the phylogenetic characteristics, origins, history, and geographical evolution of SVA on a global scale, unlocking insights into developing efficient disease management strategies.

## Introduction

Senecavirus A (SVA), also named Seneca Valley virus (SVV), is a non-enveloped, single-stranded, positive-sense RNA virus, that is the only member of the genus *Senecavirus* under the family *Picornaviridae*. SVA has a linear RNA genome of approximately 7.3 kb enclosed in an icosahedral capsid and the diameter of its viral particle is around 30 nm (Hales et al., 2008; Vannucci et al., 2015; Adams et al., 2016). Similar to other picornaviruses, the genome of SVA encodes an individual open reading frame (ORF) and the single ORF encodes a polyprotein precursor with the representative “L + 4 + 3 + 4” pattern of the *Picornaviridae*. This polyprotein is subsequently cleaved into the L (leader protein), P1 (VP4, VP2, VP3, VP1), P2 (2A, 2B, 2C), and P3 (3A, 3B, 3C, 3D) proteins (Sun et al., 2016). Representative clinical symptoms in pigs caused by SVA include high mortality in neonatal piglets (40–80% in 0–3-day-old piglets and 0–30% in 4–7-day-old piglets), skin ulceration, and vesicular lesions especially on the feet, which are quite difficult to distinguish from other vesicular diseases including foot and mouth disease and swine vesicular stomatitis (Vannucci et al., 2015; Oliveira et al., 2017). Besides pigs, cows may also serve as natural hosts of SVA and asymptomatic SVA-positive cases are emerging, making it more difficult to control the spread of SVA (Zhang et al., 2019).

SVA (SVA-001) was first recognized as a cell culture contaminant in 2002 and the first full-length SVA genome was completed in 2008 (Hales et al., 2008; Guo et al., 2020). The identification of archived virus samples of pigs with diverse clinical manifestations revealed that SVA first emerged in 1988 in the United States (Wang et al., 2016). However, there was subsequent a series of SVA outbreaks that occurred within several months beginning in late 2014, which were estimated to have impacted 80% of all pig farms in Brazil (Leme et al., 2015, 2019a,2019b; Vannucci et al., 2015). Furthermore, over 200 cases of SVA were reported in the United States Midwest from 2015 to 2017 (Zhang et al., 2015; Leme et al., 2019a,b). Meanwhile, SVA was first reported in Guangdong Province in 2015 and following isolations from clinical samples, cases of SVA have been reported nationwide since then (Qian et al., 2016; Wu et al., 2017; Chen et al., 2018; Wang et al., 2018; Zhang et al., 2019). To date, over half of Chinese provinces, autonomous regions, and municipalities (P.A.M.s) have been reported with SVA isolation (Liu et al., 2020). However, information related to SVA epidemiology in recent years has remained limited.

A few phylogenetic analyses of SVA clustering based on locations were conducted previously, yet there is still a lack of a well-systematic assortment of SVA. Additionally, the global dispersal of SVA and its origins remain still unclear. The Bayesian systematic dynamics and systematic geographics are powerful approaches for the analysis of RNA virus evolution and transmission process (Drummond et al., 2005; Drummond and Suchard, 2008; Victoria et al., 2009; Shi et al., 2010; Jin et al., 2014). Thus, in this study, the ORF sequences of SVA strains from field specimens collected by our lab and those obtained from the National Center for Biotechnology Information (NCBI) GenBank database were compiled for the purpose of a systematic SVA classification. Furthermore, the origins, transmission routes, and phylodynamics of SVA worldwide were explored with the assistance of the Bayesian dynamic and phylogeographic frameworks, to provide a more comprehensive understanding of SVA epidemiology worldwide.

## Materials and methods

### Ethics statement

All experimental protocols processed in this study were established in accordance with the guidelines of the Institutional Animal Care and Use Committee (IACUC, SCAU-AEC-2022A010) and approved by the Animal Ethics Committee of South China Agricultural University.

### Sample collection and sequencing

SVA isolation was carried out with BHK-21 cell line (stored in our lab) using Dulbecco‘s modified eagle medium (Gibco, #C11995-500CP) with 2% fetal bovine serum (Biological Industries, 04-001-1ACS) and 1% Penicillin-Streptomycin (New Cell and Molecular Biotech Co., Ltd. #C100C5) (Liu et al., 2019). Ten distinct SVA strains were successfully isolated from field samples, including from vesicular liquid and lymph nodes obtained from suspected SVA-positive pigs (Table 1). Samples were collected from 574 specimens collected from 2018 to 2021 and were processed for RNA extraction with RNAzol (Molecular Research Center, United States). Complementary DNA of SVA was acquired by reverse transcription (StarScript II MMLV, GeneStar, Beijing, China) and was amplified with polymerase chain reaction (PCR) with three pairs of self-designed primers (Table 2). The PCR amplicons were cloned into the empty pMD-18T vector followed by sequencing. All nucleotide acid and amino acid sequences were aligned, and the similarities were analyzed with MegAlign (DNAStar v7.0) (Table 3).

**TABLE 1:** Information on the 10 lab-isolated SVA strains.

Name	Sample	Location and time
SVA/China/HeN/09/2018	Vesicular liquid	Henan, Sep 2018
SVA/China/GD-HZ1/26/3/2020	Lymph nodes	Guangdong, Mar 2020
SVA/China/GD-HZ2/26/3/2020	Lymph nodes	Guangdong, Mar 2020
SVA/China/GD-SG1/27/3/2020	Lymph nodes	Guangdong, Mar 2020
SVA/China/GD-ZQ/1/4/2020	Lymph nodes	Guangdong, Apr 2020
SVA/China/GD-SG2/27/3/2020	Lymph nodes	Guangdong, Sep 2020
SVA/China/GD-SG3/2/9/2020	Lymph nodes	Guangdong, Sep 2020
SVA/China/GD-SG4/2/9/2020	Lymph nodes	Guangdong, Sep 2020
SVA/China/GD-SG5/2/9/2020	Lymph nodes	Guangdong, Sep 2020
SVA/China/GD-SG6/2/9/2020	Lymph nodes	Guangdong, Sep 2020
SVA/China/GD-SG7/2/9/2020	Lymph nodes	Guangdong, Sep 2020
SVA/China/GD-GZ/09/12/21	Lymph nodes	Guangdong, Dec 2021
SVA/China/GD/12/21	Lymph nodes	Guangdong, Dec 2021

**TABLE 2:** Primers for full-length SVA sequencing.

Primers	Primer sequence (5′-3′)	Length (bp)
SVA-1-F	TTTGAAATGGGGGGCTGG	2,696
SVA-1-R	GTCRGTGGAGTGGAACA	
SVA-2-F	CCCCTAYATCTCGCCCAG	2,738
SVA-2-R	AGCCCATGTCCACGTTGGG	
SVA-3-F	GGTCTTGCCCTAGCTGCGGT	2,270
SVA-3-R	GRGTTCTCCCAGAATCGC	

**TABLE 3:** Sequence identities (%) of different SVA clades.

	Clade ancestor	Clade I	Clade II	Clade III	Clade IV	Clade V	Clade VI	Clade VII
**Clade ancestor**								
Clade I	92.74							
Clade II	92.71	97.67						
Clade III	93.21	97.61	97.47					
Clade IV	93.22	97.23	97.18	97.55				
Clade V	93.88	96.05	96.05	96.46	96.66			
Clade VI	92.96	98.13	98.10	97.94	97.59	96.37		
Clade VII	93.21	98.06	98.23	98.08	97.73	96.58	98.63	

### Preliminary phylogenetic analysis

All SVA sequences (*n* = 250) were obtained from the NCBI GenBank database (by December 2021) and field samples collected by our lab, and strains of 100% identity and also reported from a similar date/place of collection were excluded by CD-HIT (Li and Godzik, 2006). The ORF sequences were manually trimmed using MEGA 11 containing all indels/substitutions referred to several representative SVA isolates (including the initial SVV-001 (NC011349.1), Canadian strain CAN/07-503297/2007 (MN233023.1), Thailand strain G27_SV_2/2016/Thailand (MF416218.1), China strain GD-S1/2018 (MK802892.1), etc.). All sequences were aligned *via* MAFFT v7.313 using default parameters (Katoh et al., 2002). The maximum likelihood (ML) tree was applied to the single ORF sequences using IQ-TREE v1.6.12 with the best-fit nucleotide substitution model of GTR + F + I + G4 and 1,000 bootstrap replicates (Nguyen et al., 2015; Rambaut et al., 2020; Kim et al., 2021). MAFFT, IQ-TREE, and related tools were integrated within PhyloSuite (Zhang et al., 2020).

### Time signal detection and evolutionary dynamics

To examine the time signals of the sequences after excluding recombinant strains using RDP4, Root-To-Tip (RTT) regression was employed using TempEst v1.5.3 (Martin et al., 2015; Rambaut et al., 2016). To eliminate the sample bias of global geographical analysis, we also excluded the strains from 2019 to 2021 since they were all extended from Chinese native clusters (Sun et al., 2019). Finally, the phylodynamic and phylogeographic analyses of the SVA sequences (*n* = 170) were conducted using BEAST v1.10.4 with the best nucleotide substitution model of GTR + I + G (Lanave et al., 1984; Zhang et al., 2020). The combination of best model performance was assessed through Marginal Likelihood Estimation using the path sampling/stepping-stone sampling (PS/SS) approaches in BEAUti (Table 4; Baele et al., 2012). The chain length (200,000,000) and log parameters (20,000) were set to assess the final effective sampling size (ESS) monitoring using Tracer v1.7.1 (Rambaut et al., 2018, 2020). The maximum clade credibility tree (MCC tree) was constructed through Tree Annotator v1.10.4 under the burn-in (10% of samples) followed by visualization, annotation, and elaboration using FigTree v1.4.4 (Rambaut, 2019).

**TABLE 4:** The comparison among different models (PS/SS).

Molecular clock	Tree prior	PS	SS
Uncorrelated lognormal	Constant size	−42260.29	–42313.57
	Bayesian skyline	−42225.72	–42293.51
	Exponential growth	−42237.76	–42290
Strict	Constant size	−42465.65	–42520.33
	Bayesian skyline	−42423.01	–42479.67
	Exponential growth	−42423.96	–42476.6

### Systematic Bayesian geographic reconstruction

Bayesian phylogeographic analyses of the sequences were conducted by assessing geographical information using BEAST v1.10.4 (Lemey et al., 2009; Sun et al., 2019). The Bayes Factor (BF) was calculated and visualized by comparing the posterior and prior probabilities that the individual rates were non-zero *via* spreaD3 v0.9.7.1 (Bielejec et al., 2016). The transmission routes for which BF is greater than 3 were considered to have well-supported diffusion rates, indicating well-supported migration routes between different geographical areas that constituted the SVA migration schematic diagram.

## Results

### Genomic and preliminary phylogenetic analysis

**FIGURE 1:**
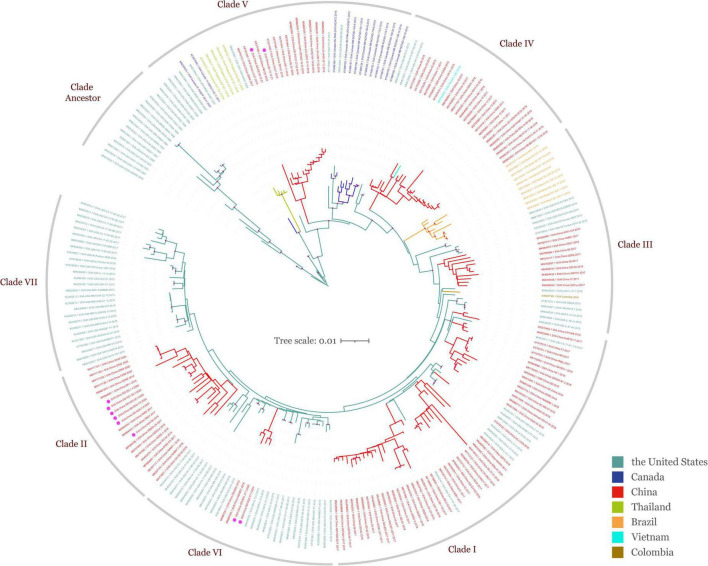
Global ML phylogeny of all publicly available SVA strains and SVA isolated in our lab based on the ORF gene. The GTR + F + I + G4 nucleotide substitution model was used for phylogenetic tree construction. All SVA strains (*n* = 250) from different countries are grouped by color and were clustered into eight clades including Clade Ancestor and Clade I–VII. The scale bar represents the number of substitutions per site along the branch in the tree topology. Light mauve dots on the tree topology indicate the bootstrap value of 100. Isolates obtained from field specimens are marked with magenta dots.

To obtain the full information and deepen our understanding of SVA, a total of 250 SVA sequences were collected from the NCBI GenBank database and field samples from our lab, and the ML phylogenetic tree was constructed after sequence alignment and pairwise comparison (Figure 1 and Table 1). Global phylogenetic analysis revealed that all representative SVA strains could be divided into eight clades: the Clade I–VII and the Clade Ancestor. The Clade Ancestor was the most initial SVA isolates and the Clade I–VII represented the circulating isolates in recent years. It could be found that Clade I–VII exhibited sequence identities in the same range (96.05–98.63%) while the Clade Ancestor displayed greater divergence (92.71–93.88%). Among Clade I–VII, Clade V showed greater divergence compared with other clades (Table 3). Furthermore, isolates from the United States were present in all clades except Clade II and IV. In China, SVA strains have been reported to have dispersed into six clades including Clade I–VI. In addition, SVA isolated from our lab were located in Clade II, V, and VI, indicating that SVA was diversified in China over recent years. Meanwhile, SVA reported from Brazil and Colombia were located in Clade III. SVA strains from Canada showed up in Clade V, and Clade V and IV SVA strains appeared in Thailand and Vietnam (Figure 1). Our findings suggested that each clade, from Clade I to VII, represented a constant genetic evolution and divergence.

### Evolutionary and population dynamics

**FIGURE 2:**
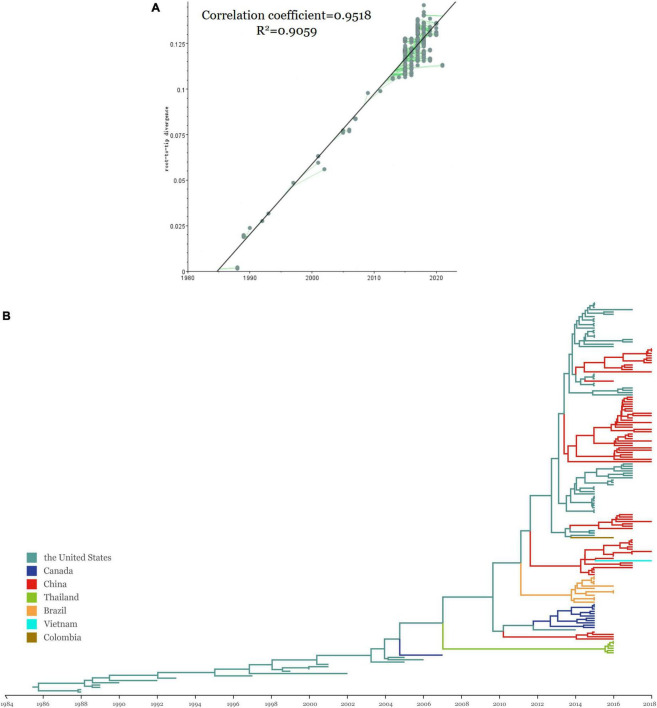
**(A)** Regression of the RTT genetic distance against the sampling time for evaluation of the molecular clock models using TempEst v1.5.3. Parameters including correlation coefficient and R2 are shown beside the linear regression. **(B)** The MCC tree of SVA strains using the ORF gene with BEAST v1.10.4 and FigTree v1.4.4. Colors represent different sampling locations, namely the United States (teal), China (red), Canada (blue), Colombia (tan) and Brazil (orange), Thailand (green), and Vietnam (purple).

The time signal was determined by the double verification of the RTT methods (Figure 2A). The RTT correlation coefficient was 0.9518 and the coefficient of determination (*R*^2^-value) was 0.9059, indicating that the time signal was of good quality. The uncorrelated lognormal relaxed molecular clock model and the Bayesian Skyline model were the most suitable models for this study after comparing different model combinations (Table 4). The topology of the MCC tree was relatively consistent with the ML tree. From the MCC tree, the United States was estimated to be the original reservoir of SVA. Furthermore, epidemic clusters of SVA developed in Thailand and Brazil developed (Figure 2B). Evolutionary analysis can provide a clear picture that the original emergence of SVA was September 1985 with the 95% highest probability density (HPD) from July 1983 to April 1987, which is around but earlier than the earliest described American case from the archived sample known of 1988. Furthermore, according to the MCC tree, the first presence of SVA in Canada, Thailand, and China was in around 2004, 2007, and 2010, respectively. The emerging time of the main clades circulating in China and the United States were as follows: Clade I: September 2012 [95% HPD (November 2011, June 2013)], Clade VI: February 2013 [95% HPD (June 2012, August 2013)], Clade VII: January 2014 [95% HPD (May 2014, September 2013)]. The average evolution rate of SVA was subsequently calculated and was inferred as 3.93 × 10^–3^ substitutions per nucleotide site per year (s/n/y) with the 95% HPD range from 3.27 × 10^–3^ to 4.68 × 10^–3^ s/n/y.

**FIGURE 3:**
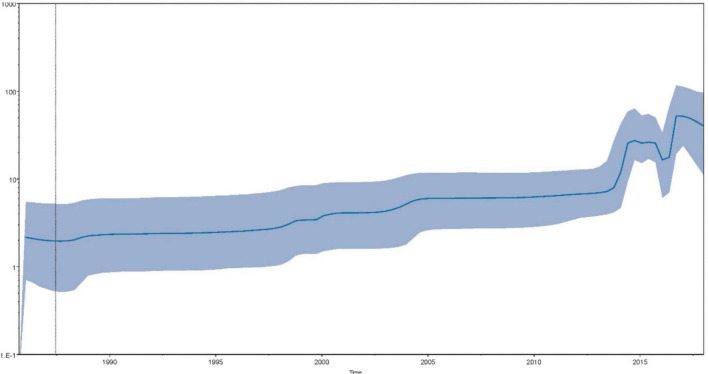
The Bayesian skyline plot depicts the temporal variation of the effective population size of SVA. The x-axis shows the time (year) and the y-axis indicates the product of the generation length in years and the effective population size. The dark blue solid line represents the median of the effective population size and the light blue shaded area indicates the 95% HPD.

Bayesian skyline plot coalescent model was processed to reconstruct the history of the effective population size and showed its temporal shifts (Figure 3; Drummond et al., 2005). Briefly, after a stably increasing period, the population size underwent two consequent rounds of spike periods since 2015. The effective population size showed an overall augmentation since the 1980s. Overall, the findings of sequence-based on the MCC tree were consistent with the results of epidemiological investigations into the timing of the initial emergence of SVA.

### Phylogeography and geographic circulation

**FIGURE 4:**
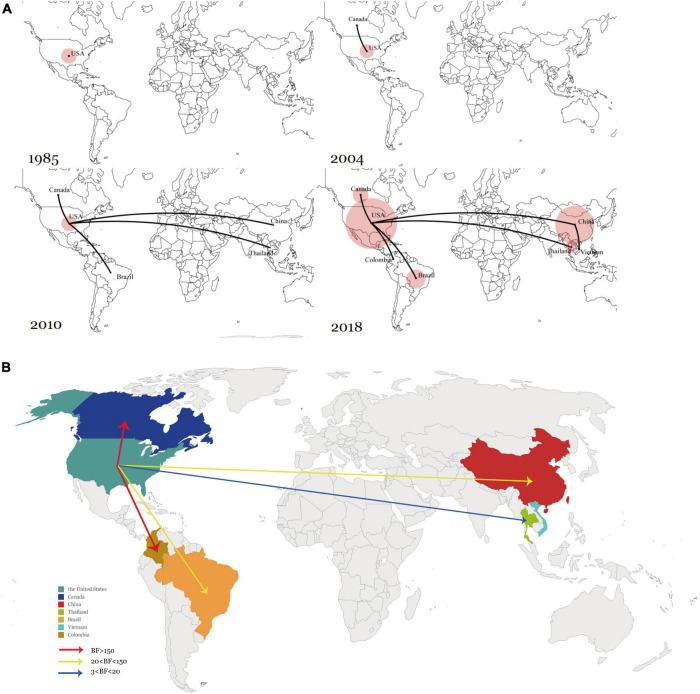
**(A)** Spatiotemporal dynamics of SVA among different regions. **(B)** Global transmission links of SVA tested by Bayes Factor for significant non-zero rates supported by the BSSVS. Line colors represent the relative strength by which the rates are supported: very strong (BF > 150, red) and strong (20 < BF < 150, yellow), and positive (BF > 3). Line directions indicate the direction of transmission.

The historical distribution of SVA was determined and visualized *via* global dynamism through maps. Conforming with the time points of the dispersal patterns, the United States was the origin of SVA, which then SVA was spread outwards to Canada in the early 2000s. Transmission to South America, China, and Southeast Asia then began in the 2010s. Since 2015, the United States and China have maintained a relatively large SVA population size, and they have also become the main SVA epidemic areas (Figure 4A).

To verify the inferred viral transmission routes, Bayesian Stochastic Search Variable Selection (BSSVS) was used to identify the most parsimonious descriptions of phylogenetic dispersal processes. The asymmetrical substitution model allowed for the BF value (> 3) determined among seven countries that had claimed SVA circulation. In summary, five transition events between discrete geographical locations were statistically well supported. Among the five transmission routes, SVA from the United States to Canada and Colombia were very strongly supported with a BF > 150 while the routes between the United States to Brazil and China exhibited 20 < BF < 150. In addition, transmission between the United States and Thailand was also statistically supported with 3 < BF < 20. Overall, the results showed that the United States had acted as the main source of SVA, and the transmission from the United States to Canada, Brazil, Colombia, China, and Thailand, was well supported by the BF values (Figure 4B).

## Discussion

Several SVA epidemics have occurred since 2015, following the initial appearances of sporadic cases. Consequently, the global swine industries have suffered tremendous losses, while new viral clades have emerged (Leme et al., 2015; Vannucci et al., 2015; Qian et al., 2016; Wu et al., 2017; Saeng-Chuto et al., 2018; Wang et al., 2018). Genomic differences gradually emerged with the continuous spread and evolution of SVA. Previously constructed phylogenies of SVA have classified SVA based on the VP1 gene, and SVA has been reported to cluster in the US-like and Canadian-like clades by location of isolation (Chen et al., 2018; Zhang et al., 2021). Moreover, another earlier phylogenetic analysis indicated that the SVA strains isolated from South China could be clustered into five distinct groups based on the VP1, 3C, and 3D genes (Sun et al., 2018; Wang et al., 2019; Liu et al., 2020). In addition, a recent paper in 2021 mentioned SVA could be clustered into two groups containing two subgroups, respectively (Jiang et al., 2021). To clarify the phylogeny of SVA, we performed an ML tree construction based on the ORF gene in this study. The ML tree was constructed using a comprehensive dataset from GenBank and strains isolated from samples collected by our lab over the last 5 years. According to the topology of the ML tree, the representative SVA strains were divided into eight clades including the Clade Ancestor representing the very first SVA cases, and Clade I–VII representing the isolates that have been circulating since the mid-2010s. The initial sequencings of SVA archived samples and outbreaks were limited by their small magnitudes. However, a much larger population size and duration of SVA with distinct epidemiological patterns were detected due to the recent outbreaks and this virus has received more attention since the 2010s. Based on our field samples of SVA-positive, the strains of SVA currently circulating in China belong to Clade II, V, and VI. The co-occurrences of United States/Brazilian in Clade III and United States/Chinese strains in Clade I were also detected. These findings suggested that SVA has diversified among different countries, thereby increasing the difficulty of controlling SVA on a global scale. More importantly, this phylogeny study utilized global SVA strains in this study rather than limited data. As the first to systematically investigate all available SVA strains into distinct clades, this study could act as a good reference in future SVA epidemiological studies.

Bayesian phylodynamic models have rarely been used to facilitate disease prevention or to control pathogens in the pig industry (Alkhamis et al., 2016). In this study, the Bayesian algorithm system was processed to improve our understanding of the global history of SVA epidemics since the employment of the system in the RNA virus control such as COVID-19 pandemic management (Sun et al., 2019; Lemey et al., 2020; Li et al., 2020; Ades et al., 2021; Kline et al., 2021; Yiannoutsos et al., 2021). From the results of phylodynamic analysis, the average evolution rate of SVA was slightly lower than previously reported, which might be attributed to the different datasets analyzed (Xu et al., 2017). Meanwhile, the viral population dynamics can be seen from the Bayesian skyline plot. Since 2015, the population of SVA increased rapidly. In 2015, SVA outbreaks began being frequently reported worldwide, including in Canada, China, Brazil, Colombia, etc. The stable SVA population size suddenly increased again in 2017. Furthermore, as the prevalence and re-emergence of SVA have increased worldwide, the monitoring of SVA was being intensified, with larger volumes of samples being screened and the SVA diagnosis optimized. A large number of strains were isolated since then, which could be a reason why the size of the SVA population quickly increased (Hause et al., 2016; Joshi et al., 2016, 2020; Houston et al., 2019; Liu et al., 2020; Wang et al., 2020). Furthermore, the Bayesian systematic analysis used in this study can be employed as a real-time early monitoring system of SVA, thus allowing the pig industry and government agencies to make reasonable decisions in a timely manner.

The main countries that reported SVA was primarily reported from the United States and Canada in 2014. During this period, only one complete strain of the virus was detected in Canada, which may have affected the structural distribution of the early branches of the MCC tree to a certain extent (Leme et al., 2017). In addition, the population conditions were unclear prior to the mid-2010s, due to a lack of sampling and limited SVA surveillance. Consequently, SVA exhibited little genetic diversity by 2014. However, with its continuous spreading and evolution, SVA after 2014 was clustered into seven clades (Clade I–VII). This genetic diversity could further enhance viral diffusion and increase the risk of exposure of pigs with the emergence of high virulent SVA, even rendering spill-over events. From the phylogeography analysis, we can see that the United States was estimated to be the origin of SVA, and then it spread to Canada around 2004, and then around 2010, SVA was spread from the United States to China and Brazil, respectively, even in the absence of shared borders. Besides, all cases of SVA (except in Vietnam) were identified to be imported from the United States based on the well-supported BF values, which is consistent with the frequent pork trade between these countries and the United States. Moreover, SVA in Thailand and Brazil imported and had already developed respective native clusters in their own countries.

This study has several limitations. First, the data for some countries, like Vietnam and Colombia, were insufficient enough. BEAST framework utilizes efficient MCMC chain statistical methods to combine molecular phylogenetic relationship reconstruction with complex discrete trait evolution, divergence time, and demographic models for inferring the phylodynamics of the SVA population in this study. However, the certainty of the output may be affected by a series of factors including the integrity of the sequence information and the size of sequence data. Therefore, a more in-depth analysis of the true transmission links associated with these nations remains to be supported by more comprehensive data (Lemey et al., 2009). Also, SVA transmission at a province/state level in China and in the United States would be interesting to study where about one-third isolates lack the information of place of collection (Supplementary Table 1). Relevant work can be further dug with greater data volume and more comprehensive/accurate isolates information. Furthermore, we are also concerned about the sampling bias in this section of selecting SVA strains prior to 2019 for the elimination of the sample bias. To examine the possible novel introductions into China, we re-inspected the strains isolated and reported after 2019. All newly isolated SVA strains were extended from China clusters through evolutionary relation. Thus, novel introductions to China after 2019 were neglected.

In summary, this study revealed for the first time the introduction and global transmission of SVA. Moreover, by processing the phylodynamic and phylogeography in the framework of Bayesian statistics can we determine the origin of SVA was the United States and the introduction of SVA in most countries was from the United States, which has also acted as the disease epicenter. The effective population size, genetic diversity, and the risk of SVA spreading to various countries have elevated over time. Our study provides fresh insights into the global dispersal of SVA, and a better understanding requires more consideration of SVA epidemiological research.

## Data availability statement

The datasets presented in this study can be found in online repositories. The names of the repository/repositories and accession number(s) can be found below: https://www.ncbi.nlm.nih.gov/genbank/, ON868368; https://www.ncbi.nlm.nih.gov/genbank/, ON868369; https://www.ncbi.nlm.nih.gov/genbank/, ON868370; https://www.ncbi.nlm.nih.gov/genbank/, ON868371; https://www.ncbi.nlm.nih.gov/genbank/, ON868372; https://www.ncbi.nlm.nih.gov/genbank/, ON868373; https://www.ncbi.nlm.nih.gov/genbank/, ON868374; https://www.ncbi.nlm.nih.gov/genbank/, ON868375; https://www.ncbi.nlm.nih.gov/genbank/, ON868376; and https://www.ncbi.nlm.nih.gov/genbank/, ON868377.

## Ethics statement

The animal study was reviewed and approved by the Institutional Animal Care and Use Committee (IACUC, SCAU-AEC-2022A010).

## Author contributions

HG and Y-JC designed the study and processed the data. HG wrote the manuscript. J-BX, Z-YX, JL, and S-JX helped, collected, and processed the data. HG, Y-JC, and Y-KS participated in the analysis and discussion. X-QX and Y-FZ helped with sample collection and diagnosis. Y-KS and G-HZ supervised and reviewed the manuscript. All authors read and approved the final manuscript.
